# Aquaporins in the Cornea

**DOI:** 10.3390/ijms25073748

**Published:** 2024-03-28

**Authors:** Samuel Melnyk, Wendy B. Bollag

**Affiliations:** 1 Department of Physiology, Medical College of Georgia, Augusta University, Augusta, GA 30912, USA; smelnyk@augusta.edu; 2 Charlie Norwood Department of Veterans Affairs Medical Center, Augusta, GA 30904, USA

**Keywords:** aquaporin (AQP), cornea, epithelium, wound healing

## Abstract

The cornea is an avascular, transparent tissue that allows light to enter the visual system. Accurate vision requires proper maintenance of the cornea’s integrity and structure. Due to its exposure to the external environment, the cornea is prone to injury and must undergo proper wound healing to restore vision. Aquaporins (AQPs) are a family of water channels important for passive water transport and, in some family members, the transport of other small molecules; AQPs are expressed in all layers of the cornea. Although their functions as water channels are well established, the direct function of AQPs in the cornea is still being determined and is the focus of this review. AQPs, primarily AQP1, AQP3, and AQP5, have been found to play an important role in maintaining water homeostasis, the corneal structure in relation to proper hydration, and stress responses, as well as wound healing in all layers of the cornea. Due to their many functions in the cornea, the identification of drug targets that modulate the expression of AQPs in the cornea could be beneficial to promote corneal wound healing and restore proper function of this tissue crucial for vision.

## 1. Introduction

The most important refractive element in the visual system, the cornea, is also the most anterior portion of the eye, making it a frontline defense against external threats. The cornea is an avascular, transparent tissue that allows light to enter the visual system. Proper vision requires proper maintenance of the cornea’s integrity and structure. Due to the cornea being exposed to the external environment, its outermost layer, the stratified squamous epithelium, has developed to not only rapidly heal wounds incurred as a result of environmental insults but also to fight the infectious agents that can be allowed entry by these wounds. Corneal wounds can occur because of injury, burns, trauma, or surgery and are painful and potentially blinding. The cornea is prone to injury because of its interface with the environment, and with any damage, there is always the risk of vision loss, whether temporary or permanent [1,2]. In addition, since visual health is critical to long-term productivity and quality of life, improving the efficacy of therapies to treat acute eye damage, promote healing, and reduce excessive inflammation, which can endanger transparency, will have profound implications for patients.

Normally, the cornea, when injured, heals rapidly, restoring its structure and integrity, but there are some individuals, such as those with diabetes, whose corneas heal slowly or not at all, which can result in major visual impairment [3,4]. It is important to understand the mechanisms via which the cornea retains and/or restores its structure and regenerates itself following injury in order to find ways to improve wound healing and identify treatments for corneal irregularities. A family of proteins that have been shown to be important for maintaining corneal structure and are involved in the process of regeneration in this tissue is the water channel family of aquaporins (AQPs). This review will focus on the AQPs found in the cornea and the roles they play in preserving corneal function in terms of both overall structure and wound healing/regeneration.

## 2. Basic Histology and Function of the Different Layers of the Cornea

The cornea is the anterior portion of the eye and protects the inner eye from the external environment [5]. It consists of five distinct layers: the epithelium, an anterior limiting basement membrane (Bowman’s membrane), the stroma, a posterior limiting basement membrane (Descemet’s membrane), and the endothelium. The epithelium constitutes approximately 10% of the corneal thickness and comprises the non-keratinized squamous epithelium. This layer is highly proliferative, a characteristic that is important for cell renewal and repair of the epithelium, which is about five or six cell layers in thickness. The corneal epithelial layer serves as a protective barrier against the external environment and, through its regenerative properties, allows healing from multiple types of injuries that can occur as a result of physical abrasion, burns (chemical and thermal), and corneal surgery. Corneal wounds are extremely painful and can predispose an individual to infection but usually heal rapidly due to the highly regenerative properties of the cornea. In contrast, the stroma constitutes about 90% of the cornea and is a connective tissue made up fundamentally of an extracellular matrix and a small population of keratocytes. In addition, the stroma contains a large number of hydrophilic glycoproteins, creating a water gradient that promotes an influx of water into the endothelium from the aqueous humor; AQPs, and to a certain extent tight junctions, act as passive transport mechanisms for this influx.

The endothelium is critically involved in maintaining the hydration state of the corneal stroma via a “pumping” mechanism that removes water from the stroma. The mechanism consists of multiple ion-transport channels dependent on the active transport of sodium and potassium by Na^+^/K^+^ ATPase-2 and the passive transport of water through aquaporin 1 (AQP1), as will be discussed below [6,7,8]. Thus, to maintain proper hydration and prevent edema, the endothelium “pumps” water out through this system of transporters and channels. This mechanism ultimately results in the shuttling of bicarbonate and lactate from the stroma to the anterior chamber, creating an osmotic gradient for water to travel from the stroma to the anterior chamber (as reviewed in [6,7,8]). By preventing edema and the accompanying swelling of the cornea, the endothelial layer helps to preserve corneal clarity and visual acuity [9,10,11,12].

Corneal blindness is the fourth leading cause of blindness worldwide [11]. Blindness from pathological conditions of the cornea affects the productivity and quality of life of close to 5 million people around the world [11]. As the boundary between the internal and external environment, the cornea is prone to injury [2]. The most common injuries in the eye are from foreign objects, chemicals, and scratches of the cornea. These injuries, as well as ocular surface diseases such as dry eye disease (DED) and pterygium, can decrease the quality of life of an individual and result in visual impairment or blindness [2]. The financial cost associated with ocular surface disease has been an increasing burden on the healthcare system and could worsen, as the emerging epidemic of diabetes may lead to delayed corneal wound healing, resulting in increased ophthalmologic treatments and lost work [3,4,13,14].

## 3. The Corneal Wound Healing Process

Corneal wound healing is a multistep process involving apoptosis, cellular migration, proliferation, differentiation, and extracellular matrix interactions at the wound site; it is regulated by various inflammatory mediators and growth factors. This process has been reviewed many times, and for further information, the following articles are suggested [5,12,15]. However, it should briefly be noted that there are differences in how each corneal layer regenerates itself. Epithelial wounds regenerate as a result of the migration, proliferation, and differentiation of limbal stem cells [15]. In contrast, stromal wounds heal following the transformation of stromal keratocytes to fibroblasts and myofibroblasts that remodel the damaged stroma, together with the participation of resident and circulating immune cells; these myofibrocytes subsequently undergo apoptosis, and their excessive and/or continuing activation can lead to corneal haze [15]. The endothelium heals via cell migration and spreading, with cell proliferation playing only a secondary role. The regeneration of the cornea to replenish missing or damaged tissues as a result of injury is important to maintaining vision by repairing the corneal shape/structure in order to preserve transparency. An important aspect of corneal function, including in the wound healing process, is the activity of aquaporins.

## 4. Aquaporin Structure and Function

**Figure 1:**
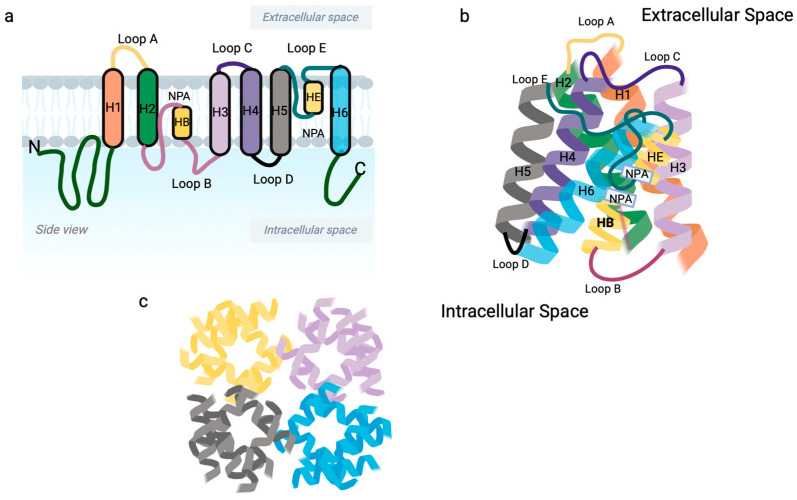
General topology and structure of aquaporins. (**a**) Each AQP is composed of six membrane spanning α-helices (H1–6) and five connecting loops (A–E) with two long loops: an extracellular (HE) and a cytosolic loop (HB) that come together to help to form the pore. (**b**) Side view of a AQP monomer illustrating that the loops of the pore each contain an NPA (asparagine–proline–alanine) motif that provides the channel’s water selectivity filter. (**c**) Top view of a tetramer of AQP, with each monomer exhibiting a functional water pore. Created with BioRender.com; accessed 19 February 2024.

Aquaporins (AQPs) are a family of water channels that are conserved throughout all kingdoms of life. In mammals, the AQP family consists of 13 members (AQP0–AQP12) that transport water; some members also transport other small non-charged solutes. Thus, AQPs are separated into two classes: those that transport water only (AQP0, AQP1, AQP2, AQP4, AQP5, AQP6 and AQP8) and the aquaglyceroporins that also transport small solutes like hydrogen peroxide and glycerol (AQP3, AQP7, AQP9 and AQP10) [16,17,18,19]. AQPs are small membrane proteins (about 270 amino acids) that are composed of six membrane-spanning α-helices and five connecting loops (A–E) with two long loops: an extracytosolic (E) and a cytosolic loop (B) meeting together to help to form the pore [16,17,18,19,20] (Figure 1). The loops of the pore each contain an NPA (asparagine–proline–alanine) motif that functions to provide the channel’s water selectivity filter and appears to promote plasma membrane localization of at least some AQPs, like AQP4 [21].

Aquaporin transport activity can be regulated by various factors. The pH can affect the permeability of water and glycerol through several of these channels. For example, at neutral pH, AQP3 is permeable to water and glycerol but at a pH of less than 6, minimal permeation of either water or glycerol is observed [17]. In contrast, AQP6 is only active at a pH of less than or equal to 5.5, while AQP1 is not sensitive to pH (reviewed in [17]). Hormonal signaling, such as that induced by the binding of vasopressin, also known as antidiuretic hormone, to its receptor increases the cellular water permeability of AQP1, AQP2, and AQP4 through the redistribution of these AQPs from storage vesicles to the plasma membrane. The binding of calcium to the AQP0 protein, as well as the phosphorylation of APQ4, have also been observed to regulate water permeability (reviewed in [17]). In a colon cell line, AQP3 has been demonstrated to traffic, in response to epinephrine, from the cytosolic fraction to the plasma membrane, through the action of protein kinase C [22]. AQPs can also be glycosylated at asparagine 42 via N-linked glycosylation [19]. In yeast expressing human recombinant AQP10, the glycosylation of AQP10 helps to increase its thermostability [23]. AQP2 has been found to tetramerize in the ER, and N-linked glycosylation is important for its transport from the Golgi complex to the plasma membrane in the Madin–Darby canine kidney type-I cell line [24]. The effect of glycosylation for all AQP family members with regard to function and localization, however, has not been extensively studied.

## 5. Aquaporins in the Cornea

AQPs are conserved throughout the animal kingdom, although there are some differences in the AQPs found in the corneas of various species. It was previously thought that AQP4 was not expressed in the human cornea [25]. However, its expression was later detected in endothelial cells of normal corneas [26], whereas in stromal cells of diseased corneas [with pseudophakic bullous keratopathy (PBK)/aphakic bullous keratopathy (AKB), see Section 5.2)], AQP4 staining was increased concomitantly with its general upregulation [26]. Nevertheless, the function of AQP4, in either normal or diseased corneas, is unknown [26]. Furthermore, AQP4 expression in the cornea is not conserved throughout the animal kingdom, since it has not been found in the cornea of rats [27], dogs [28], rabbits [29], mice [30], or cows [31]. On the other hand, AQP11 was found to be expressed via Western blotting in rat cornea but was not detected with immunostaining [32]. Using RT-PCR analysis and immunohistochemical staining, Tran et al. [33] found that AQP11 is expressed in the basal cells of the epithelium in the limbal area of the human eye. AQP7 was found in the cytoplasm of basal cells in the anterior corneal epithelium, the conjunctival epithelium in the limbal region, and the corneal endothelium in human corneas [34]. Although these APQs have been found to be expressed in the cornea, they have not been extensively studied, and their function is not completely understood.

**Figure 2:**
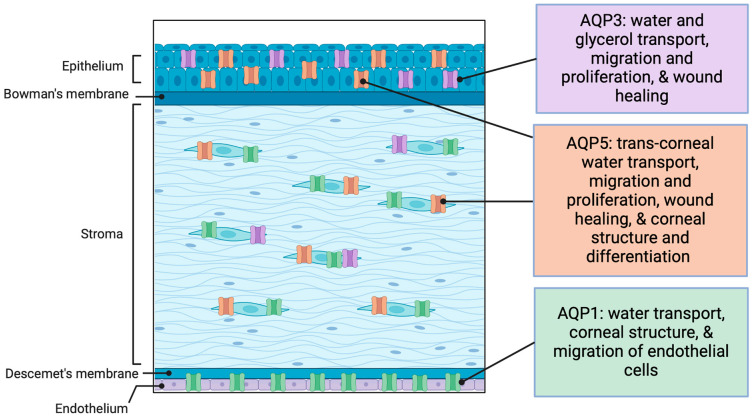
The locations of aquaporins (AQPs) with known functions in the cornea and their general roles. AQP1 is located primarily in the endothelium, where it plays a role in the water transport important for maintaining the proper hydration of the cornea. AQP1 also contributes to the migration of endothelial cells and the migration and proliferation of stromal keratocytes. AQP3 is primarily located in the epithelial and stromal cells and is important for glycerol and water transport to play a part in migration and proliferation for wound healing. AQP5 is important for trans-corneal water transport, migration and proliferation, corneal structure, and differentiation. AQP5 is primarily found in cells in the epithelium and stroma. Created with Biorender.com; accessed 19 February 2024.

In mammals, the main AQPs with conserved expression in the cornea of different species are AQP1, AQP3, and AQP5, which are the focus of this review, as they are the best-studied corneal AQPs with described functions (Figure 2). AQP1 is mainly found in the endothelium with some expression in stromal cells, and this expression pattern is conserved in mice, pigs, cows, goats, horses, dogs, cats, humans, rabbits and rats [25,27,28,29,31,32,35,36,37]; AQP5 is found in the corneal epithelia of pigs, cows, goats, horses, dogs, cats, humans, rabbits, mice and rats [20,25,27,28,29,31,32,35,36,37]. AQP3 is expressed predominantly in the corneal epithelium (in the basal and suprabasal cells) and in small amounts in the corneal stromal cells in humans, rats, dogs, mice, and rabbits [25,26,28,38,39]. The functions of AQP1, AQP3, and AQP5 in the cornea appear to involve the general maintenance of corneal structure, as well as corneal wound healing and repair.

### 5.1. AQP5

*AQP5* was discovered and cloned from salivary gland cDNA and, through Northern blot analysis, found to be expressed in the salivary gland, the lacrimal gland of the eye, the lung, and the trachea [20]. AQP5 expression is also enriched in the cornea and localizes specifically to the corneal epithelium [25,27,40]. In global *AQP5* knockout mice in vivo, the ablation of *AQP5* leads to an increase in corneal thickness, which is further enhanced as the mice age, as well as a reduction in the extent of corneal swelling when a hypotonic solution is introduced [37,41]. Verkman’s laboratory additionally showed that AQP5 is a principal route of water flux across the intact corneal epithelium: the deletion of *APQ5* caused an approximate five-fold reduction in trans-corneal water flux through intact corneas, which could be restored via the removal of the epithelium [30]. *AQP5* knockout mice, but not transgenic mice lacking *AQP1* or *AQP3*, were found to exhibit tear film hypertonicity, likely due to reduced trans-corneal water secretion in response to evaporative water loss [42]. In fact, in keratoconus, an eye disorder in which the cornea thins and gradually bulges outward, *AQP5* was found in an RNA-sequencing analysis to be the most downregulated gene when comparing multiple diseased corneas to control corneas (*AQP3* was also found to be downregulated in keratoconus but not to as great an extent as *AQP5* [43]). On the other hand, another study by Garfias et al. [44] found that differences in AQP5 mRNA and protein levels were not associated with keratoconus, and no alterations in the location of these proteins were observed when analyzing normal in comparison with diseased corneas.

One of the pathways via which AQP5 can be regulated is the cAMP-dependent protein kinase (PKA) signaling pathway through the PKA-mediated phosphorylation of threonine 259; however, the exact function of this post-translational modification is currently unknown [10,45,46]. Kumari et al. [47] found that AQP5 in the cornea can be phosphorylated; these authors further determined that the exposure of mouse corneas to PKA inhibitors ex vivo resulted in an increase in AQP5 plasma membrane localization and abundance in the epithelium. In contrast, the use of membrane-permeable cAMP induced a reduction in the plasma membrane localization of AQP5 in the epithelium [47]. Presumably, such localization is important in AQP5′s water flux capacity. Another pathway that was found to affect the regulation of AQP5 in an immortalized human corneal epithelial cell line was the JNK1/2 MAPK signaling pathway under hyper-osmotic conditions, in which inflammation and caspase-1 expression are increased, as are phosphorylated JNK1/2 and AQP5. Upon the inhibition of JNK1/2, AQP5 protein levels, cell death, and inflammatory markers are all reduced [48]. In fact, siRNA knockdown of AQP5 under hyper-osmotic conditions also reduces cell death and inflammatory markers, indicating that AQP5 is important for stress responses under hyper-osmotic conditions and suggesting that this channel is regulated through JNK signaling [48].

AQP5 has been found by Kumari et al. [49] to promote wound healing in the cornea by facilitating cell migration and proliferation. Upon shock blast wounding, AQP5 expression was found to be induced, and its localization was changed [50]: in addition to an increase in AQP5 expression, a shift in localization to the basally located epithelial cells was observed in comparison to uninjured corneas, where the expression pattern was found to be more of mixed membrane and cytoplasmic localization. However, why the shift occurs is still to be determined [50]. AQP5 might also be important for corneal innervation, as Liu et al. [51] demonstrated that AQP5 facilitates corneal epithelial wound healing and nerve regeneration through its activation of the AKT signaling pathway mediated by increased nerve growth factor (NGF) expression. These authors found that upon the wounding of wild-type corneas, AQP5 is upregulated, which leads to an increase in NGF expression that is less pronounced in *AQP5* knockout mice [51]. These authors further demonstrated that the treatment of wounded *AQP5* knockout mice with NGF results in the recovery of the observed reduction in nerve regeneration and AKT phosphorylation/activation. Furthermore, when experiments were repeated in the presence of AKT inhibitors, NGF treatment is unable to restore corneal nerve fiber density and sensitivity in the *AQP5* knockout mice [51]. In aged mice (older than 6 months), *AQP5* knockout leads to significantly more corneal neovascularization, inflammatory cell infiltration, and corneal haze [41]. Aged AQP5 knockout mice show a reduction in Wnt signaling, which is important for differentiation and migration during development [41]. The introduction of IIC3 (an inhibitor of the Wnt antagonist Dickkopf WNT Signaling Pathway Inhibitor 1, or DKK1) restores the wound healing rate of *AQP5* knockout mice to that of the wild type, indicating that AQP5 plays an important role in activating Wnt signaling in the cornea, although the method of activation is still unknown [41]. AQP5 may also play a role in the differentiation of corneal epithelium cells, since aged *AQP5* knockout mice show a reduction in the mRNA and protein levels of keratin 12, a marker of the differentiated corneal epithelium, as well as an increase in keratin 1, 10 and 14 mRNA and protein levels, which are keratins expressed by epidermal keratinocytes of the skin epithelium [41].

Ophthalmic agents used for eye disorders may also work, at least in part, by regulating AQP5. For example, gabapentin, a structural analog of gamma–amino butyric acid, has been shown to provide pain relief and have anti-inflammatory properties in glaucomatous patients [52,53], and in rabbits, the topical application of gabapentin to the eye has analgesic but not anesthetic effects [54]. Cammalleri et al. [54] found that gabapentin increases lacrimation through the upregulation of acetylcholine and norepinephrine levels and the induction of AQP5 expression in the lacrimal gland of rabbit eyes. These authors further showed that gabapentin also increases AQP5 expression in human corneal endothelial cells. This effect seems to be mediated by PKA, as gabapentin increases the phosphorylation of PKA, and phosphorylated PKA has been known to regulate AQP5 expression, as well as localization, as mentioned above [54].

### 5.2. AQP1

AQP1 is expressed in corneal keratocytes and the endothelium [25,31,35] and seems to be important for maintaining corneal structure. AQP1 deletion leads to a reduction in corneal thickness, as well as a decrease in the rate of corneal swelling when a hypotonic solution is introduced into the eye [37]. In addition, *AQP1* knockout mice treated with a hypotonic solution also exhibit a significant reduction in the rate of recovery of corneal transparency, indicating that AQP1 is involved in corneal water permeability and extrusion of fluid from the corneal stroma across the corneal endothelium [37]. This idea has been investigated further in cultured *APQ1* null mouse corneas, which were found to exhibit normal fluid transport but impaired water permeability and volume regulation following hypo-osmotic challenge [55]. However, other experiments indicate that the genetic deletion of *AQP1* in *AQP1* knockout mice does not affect osmotic water movement across intact corneas compared to wild-type mice [30].

PBK/ABK and Fuchs’ dystrophy are relatively common corneal diseases, characterized by corneal endothelial cell dysfunction and chronic edema as major components of the pathological process. In these disorders, fluid accumulates within epithelial cells (microcysts), and corneal thickness can increase by up to 100 to 300 μm, leading to loss of transparency and decreased vision [26]. AQP1 has been shown to be downregulated in Fuchs’ dystrophy and PBK/ABK corneas, which is in line with the findings of Verkman and colleagues that AQP1 knockout reduces corneal fluid elimination [26,56]. Corneal edema is also observed with corneal injuries from blast shocks [57]. These shock blast-related injuries result in increased AQP1 mRNA and protein levels, but this effect could be compensatory due to injury; however, why this occurs has not yet been further investigated [50].

AQP1 may also be important in maintaining stem cell clusters in the limbus. Higa et al. [58] found that large dendritic AQP1-positive stromal-like niche cells exist beneath the N-cadherin-positive limbal basal progenitor/stem cell clusters; however, the interaction with the stem cells, as well as the role of these AQP1-positive cells, is currently unknown. In stromal keratocytes, AQP1 has been found to accelerate cell migration in culture, as well as upon wounding in vivo [59]. Finally, AQP1 knockdown decreases cellular migration and proliferation in transformed human corneal endothelial cells [60]

As with gabapentin and AQP5, an agent used for an ophthalmic indication may also function through effects on AQP1. Thus, fibroblast growth factor 10 (FGF10) improves corneal endothelial wound healing in rabbits by inhibiting the endothelial to mesenchymal transition, which is known to be associated with scarring, as this transition results in the fibrotic transformation of corneal endothelial cells. FGF10 also reduces inflammatory markers and improves mitochondrial health [61,62]. Interestingly, in endothelial cells, FGF10 increases the protein levels of AQP1, as well as the activity of the Na+/K+-ATPase; both of these are important in the function of the corneal endothelium in terms of maintaining the cornea’s hydration, structure and shape [62].

### 5.3. AQP3

AQP3 is an aquaglyceroporin shown to transport water, hydrogen peroxide, and glycerol [40,63,64,65,66,67,68]. AQP3 is expressed in the epithelium of the cornea [25], mainly in the basal cells, but expression is also observed in some stromal cells [26]. AQP3 is also expressed in the conjunctiva [30]. *AQP3* knockout mice show no alterations in corneal epithelial thickness, morphology, or glycerol content but demonstrate a reduction in both water and glycerol transport [69]. Although AQP3 has not been found to play an important role in general water homeostasis to maintain proper structure of the cornea, AQP3 is important for the migration and proliferation of corneal epithelial cells after wounding. Indeed, *AQP3* deletion results in a delay in the healing process after wounding. This result indicates that AQP3 is a key molecule for normal cell migration and proliferation during corneal wound healing [69]. Evidence suggests a potential role for AQP3′s water transport function in cell migration [9] and its glycerol transport for its effects on proliferation. Thus, it is thought that glycerol’s effect on proliferation potentially results from actions to increase ATP levels [70] and/or its conversion to phosphatidylglycerol (PG) through the phospholipase D2 (PLD2) co-localized with AQP3 [71] (see below). AQP3 expression may also be related to corneal disease since in PBK corneas, and Fuchs’ dystrophy AQP3 is reportedly upregulated compared to normal corneas [26]; however, the reason for this change is unclear.

An important aspect of AQP3 is its colocalization with the phospholipid-metabolizing enzyme phospholipase D2 (PLD2) in the corneal epithelium [39,72], as in the epithelium of the skin [66,71,73]. In addition to the fact that AQP3 and PLD2 are co-localized, another similarity between cornea and skin is the fact that *AQP3* knockout mice demonstrate a slower rate of skin wound healing, in addition to their delayed corneal healing [69]. Not only do AQP3 and PLD2 exhibit colocalization, but they can also be co-immunoprecipitated from corneal and skin epithelial cells, indicating their physical association. As a result of this colocalization/association, the glycerol transported by AQP3 can be provided to PLD2, which can convert the glycerol to phosphatidylglycerol (PG), which serves as a lipid signal [39]. Topical treatment with the specific PG dioleoylphosphatidylglycerol (DOPG) has been shown to improve corneal wound healing not only in wild-type mice but also in *AQP3* knockout mice with delayed healing, providing evidence of the importance of the AQP3/PLD2/PG pathway in this process [39].

Finally, AQPs may not be the only conduit for water movement in the cornea. Vilas et al. [74] found that SLC4A11, a member of the solute carrier 4 family of bicarbonate transporters, can act as a water channel: these investigators observed osmotic gradient-driven cell swelling in *Xenopus laevis* oocytes and HEK293 cells expressing SCL4A11, AQP1 and NIP5;1 (a plant water and borate channel) or hCNT3 (a human nucleoside transport protein). These authors further found that *SLC4A11* knockout mice develop hazy corneas, corneal edema, and distorted endothelial cell morphology, as indicated by large swollen endothelial cells with indiscernible cell boundaries, indicating the importance of SLC4A11 in maintaining corneal structure and water transport [74]. Thus, SLC4A11 seems to be involved in forming a basolateral trans-endothelial cell water pathway that is required for fluid reabsorption from the corneal stroma, working in concert with apical AQP proteins [74] (Table 1). Indeed, mutations of the *SCL4A11* gene have been found to underlie some cases of Fuchs’ dystrophy [75], congenital hereditary endothelial dystrophy [76,77], and Harboyan syndrome [78].

## 6. Conclusions

**Figure 3:**
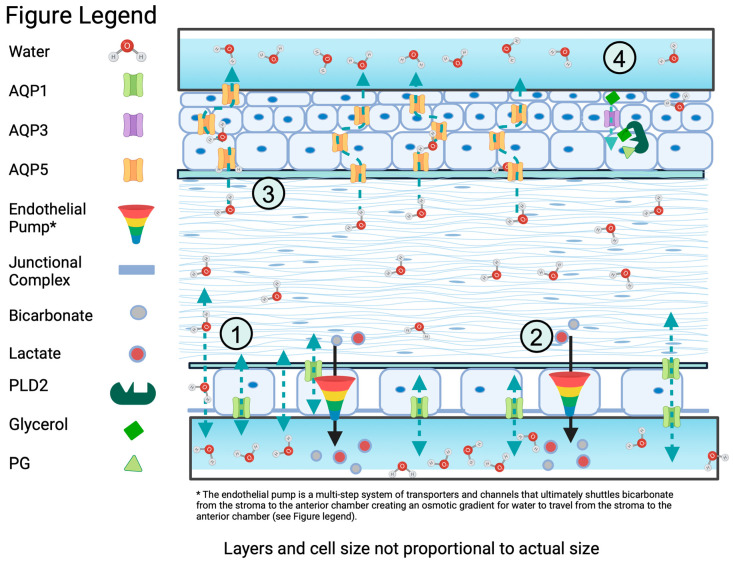
Water flow in the cornea in relation to aquaporins (AQPs). (1) Hydrophilic glycoproteins in the stroma cause water influx into the endothelium from the aqueous humor, with AQP1, and to a certain extent tight junctions, acting as passive transport mechanisms. (2) To maintain proper hydration and prevent edema, the endothelium “pumps” water out through multiple transporters and channels, which ultimately results in the shuttling of bicarbonate and lactate from the stroma to the anterior chamber, thereby creating an osmotic gradient for water to travel from the stroma to the anterior chamber (as reviewed in [6,7,8]). (3) AQP5 also shuttles water across the epithelium from the stroma to the tear film to help maintain corneal structure. (4) Although AQP3 has not been found to play an important role in water homeostasis to maintain proper structure of the cornea, AQP3 is important for the migration and proliferation of corneal cells after wounding. Evidence suggests a potential role for AQP3′s water transport function in cell migration [9] and its glycerol transport for effects on proliferation. It is thought that glycerol’s effect on proliferation potentially results from actions to increase ATP levels [70] and/or its conversion into phosphatidylglycerol (PG) through the phospholipase D2 (PLD2) co-localized PLD2 with AQP3 [71]. Created using Biorender.com; accessed 19 February 2024.

Both the structure and proper healing of the cornea are very important for maintaining visual acuity. AQPs have been found to be important not only for maintaining proper corneal hydration, which is important for the shape of the cornea but also for improving wound healing in the cornea (Figure 3). Finding targets for compounds that modulate AQP expression in the cornea might be beneficial for promoting the healing of corneal wounds, thereby helping to improve outcomes for better vision after abrasion, trauma, chemical burn, surgery, or other injuries of the cornea that result from its constant interface with the external environment.

## Figures and Tables

**Table 1:** The multiple known functions of aquaporins in the cornea.

Aquaporin *	Location in Cornea	Function	Specific Function	References
AQP5	Epithelium and Keratocytes	Water homeostasis	Trans-corneal water transport; KO leads to reduced trans-corneal water flux, tear film hypertonicity and hypotonicity-induced swelling	[30,42]
		Corneal structure	Epithelial cell proliferation and differentiation; KO leads to increased corneal thickness and decreased keratin 12 expression	[30,37,41]
		Wound healing	Cell migration, proliferation and differentiation in epithelium and keratocytes; KO results in decreased corneal nerve regeneration	[49,51,60]
		Stress responses	Induction of apoptosis with hypertonicity; KD decreases hypertonicity-induced apoptosis	[48]
		Aging-related corneal changes	Neovascularization and corneal haze; KO leads to increased neovascularization and corneal haze	[41,51]
AQP3	Epithelium and Stromal Cells	Water homeostasis	Increased water and glycerol transport; KO results in reduced water and glycerol transport	[25,69]
		Wound healing in the corneal epithelium	Cell migration and proliferation; KO leads to decreased cell migration and proliferation and delayed wound healing	[39,69]
AQP1	Endothelium and Keratocytes	Water homeostasis	Corneal water efflux; KO mice exhibit delayed recovery of hypotonicity-affected corneal transparency/reduced water extrusion	[37,55]
		Corneal structure	KO results in decreased corneal thickness and hypotonicity-induced corneal swelling	[37,55]
		Wound healing	Cell migration; KO leads to reduced stromal keratocyte migration upon wounding	[42]
SLC4A11	Endothelium	Corneal structure	KO in endothelial cells results in corneal edema and distorted cell morphology	[74]
		Water homeostasis	Trans-endothelial water transport	[74]

* AQP4 and AQP7 have only been found to be expressed in human, and AQP11 in rat and human, but there has been little research into their roles in the cornea, and their general function in this tissue is unknown [26,32,34].
